# EFFECTS OF PHYSICAL ACTIVITY ON HEALTH AMONG INFORMAL CAREGIVERS OF INDIVIDUALS WITH DEMENTIA: A META-ANALYSIS

**DOI:** 10.1093/geroni/igae098.2273

**Published:** 2024-12-31

**Authors:** Sangwoo Ahn, Misook Chung, Scott Crouter

**Affiliations:** Pennsylvania State University, Knoxville, Tennessee, United States; University of Kentucky, Lexington, Kentucky, United States; University of Tennessee Knoxville, Knoxville, Tennessee, United States

## Abstract

Caregiving for individuals with dementia, including Alzheimer’s disease and related dementias (ADRD), negatively affects informal caregivers’ health. Our review aimed to systematically evaluate the effects of physical activity on physical and psychological health and well-being among informal caregivers of individuals with ADRD because no comprehensive evidence has been drawn so far. To identify appropriate randomized controlled trials, nine databases published by July 2023 were searched. Twelve studies were included (n = 1,163 total participants; mean age = 64 years), two of which were rated as strong in methodological quality, and one as weak with the other nine showing moderate quality. Meta-analysis was performed using STATA (18.0) with the standardized mean difference (SMD; Hedge’s g) used to calculate the effect size of interventions. Physical activity, consisting of walking, Tai Chi, or multicomponent activities (aerobic, strengthening, stretching, and/or balance activities), exerted beneficial effects on static balance as an element of physical health (SMD = 0.45; p < 0.001) and psychological well-being as an element of well-being (SMD = 0.46; p < 0.001), as well as general physical health (SMD = 0.41; p = 0.03) and well-being (SMD = 0.41; p < 0.001) with moderate effect sizes. Health care providers may suggest informal caregivers engage in physical activity to improve physical and mental well-being, and further, potentially decrease some of the onerous aspects of caregiving. Given the limited number of studies in our review, future research is warranted to identify the optimal type and dose of physical activity for informal caregivers’ health.

